# The effect of air pollutants on birth weight in medium-sized towns in the state of São Paulo☆

**DOI:** 10.1016/j.rpped.2014.06.003

**Published:** 2014-12

**Authors:** Veridiana de Paula Santos, Andréa Paula Peneluppi de Medeiros, Thaiza Agostini Córdoba de Lima, Luiz Fernando Costa Nascimento

**Affiliations:** Universidade de Taubaté (UNITAU), Taubaté, SP, Brazil

**Keywords:** Low birthweight, Air pollution, Logistic regression, Linear regression

## Abstract

**OBJECTIVE:**

To investigate the effect of air pollution on birth weight in a medium-sized town in the State of São Paulo, Southeast Brazil.

**METHODS:**

Cross-sectional study using data from live births of mothers residing in São José dos Campos from 2005 to 2009. Data was obtained from the Department of Information and Computing of the Brazilian Unified Health System. Air pollutant data (PM_10_, SO_2_, and O_3_) and daily averages of their concentrations were obtained from the Environmental Sanitation & Technology Company. Statistical analysis was performed by linear and logistic regressions using the Excel and STATA v.7 software programs.

**RESULTS:**

Maternal exposure to air pollutants was not associated with low birth weight, with the exception of exposure to SO_2_ within the last month of pregnancy (OR=1.25; 95% CI=1.00-1.56). Maternal exposure to PM_10_ and SO_2_ during the last month of pregnancy led to lower weight at birth (0.28g and 3.15g, respectively) for each 1mg/m^3^ increase in the concentration of these pollutants, but without statistical significance.

**CONCLUSIONS:**

This study failed to identify a statistically significant association between the levels of air pollutants and birth weight, with the exception of exposure to SO_2_ within the last month of pregnancy.

## Introduction

Air pollution is currently one of the main public health problems, affecting the health of human beings, animals, and plants. The rapid technological advance of the modern world has resulted in an increase in the quantity and variety of pollutants eliminated in the atmosphere, affecting the quality of life on the planet.^1^ The main air pollutants in cities are particulate matter (PM_10_), ozone (O_3_), sulfur dioxide (SO_2_), carbon monoxide (CO), and nitrogen oxides (NO_2_).

Exposure to air pollutants has shown to be associated with several deleterious effects to health, even at levels considered safe by environmental legislation.^1^^,^ 2 When measuring the concentration of air pollutants in a given location, it can be identified that higher concentrations result in adverse health effects, such as an increase in the number of hospital admissions, increase in mortality, and decreased life expectancy.^3^ The effects of air pollution on outcomes related to pregnancy have also been considered in some studies.^4^^-^ 6 Among these outcomes is low birth weight (LBW),^7^^,^ 8 defined as a live birth weighing less than 2,500g.^9^ The biological mechanisms involved in fetal growth associated with environmental pollution seem related to placental changes, with anatomopathological and morphometric changes,^10^ placental infarction,^11^ and chronic villitis.^12^

A study conducted by Perera *et al* in Dominican and African-American pregnant women aged 18 to 35 years of age who had lived for at least one year in New York, who were nonsmokers without diabetes or hypertension and had negative serology for human immunodeficiency virus, indicated that in the population studied, the fetus and the newborn are more susceptible than adults to toxic environmental substances.^13^

Birth weight is an important determinant of neonatal morbimortality and post-neonatal mortality,^14^ and thus is of great importance in public health. Therefore, the World Health Organization (WHO) considers LBW as the single most important factor in child survival. Children with low birth weight are at significantly higher risk of mortality than children with birth weight ≥2,500 g.^15^ LBW is observed in 15.5% of all births worldwide. 

However, the problem does not occur uniformly among different locations, but rather is related to socioeconomic status. The highest percentage of children with LBW is concentrated in two regions of the world, Asia and Africa, with 27% and 22% of all live births showing low birth weight, respectively.^16^ In developed countries, in general the proportion of LBW is between 4% and 6%.^17^ In 2008, Brazil had a proportion of 8.3% and the city of São José dos Campos, 9.1%.^18^

LBW has been the subject of several epidemiological studies^4^^,^ 7 ^,^ 8 ^,^ 15 aiming to identify its risk factors, in an attempt to develop interventions that can reduce these rates and prevent its occurrence. The importance of LBW for public health is determined not only by the subsequent risk of mortality and morbidity, but also by the frequency at which it occurs. In this context, the present study aimed to evaluate the effect of air pollution on birth weight of newborns of mothers living in São José dos Campos, state of São Paulo, Brazil, in the years 2005-2009. 

## Methods

This was a cross-sectional study of data on all births to mothers living in the city of São José dos Campos in the years 2005 to 2009, which had completed a live birth certificate.

São José dos Campos is located 80 km from São Paulo, between the Serra do Mar and Mantiqueira mountain range, and has a population of approximately 700,000 inhabitants. Its altitude is 600m above sea level and it is located at latitude 23°11' south and longitude 45°53' west. It has approximately 1,100 industries, especially automotive, pharmaceutical, aerospace, and oil refining. 

Information on live births was obtained from the Live Birth Information System (SINASC) database through live birth certificates (LBCs), provided by the Department of Informatics of the Unified Health System (DATASUS), available at http://www.datasus.gov.br. It included infants born at term, weighing between 1,000g and 4,500g, the result of a single pregnancy, with maternal age between 20 and 34 years, after seven or more prenatal consultations and maternal level of schooling of eight or more complete years. These criteria were adopted to eliminate situations of greater vulnerability, such as preterm newborns and those weighing less than 1,000g, for which other risk factors might be more relevant than air pollution. 

The pollutants studied were PM_10_, SO_2_, and O_3_, which are quantified daily by Companhia de Tecnologia de Saneamento Ambiental (CETESB) of São José dos Campos. In Brazil, the air quality standards have been established by CONAMA Resolution No. 3/1990 (Table 1);^19^ however, in the state of São Paulo, the State Decree No. 59113, published on 04/23/2013,^20^ established new standards for air quality (Table 2). 

**Table 1:**
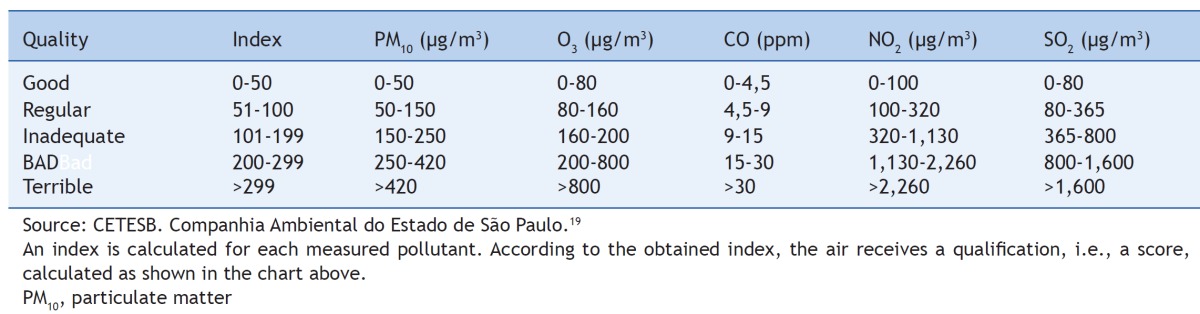
National standards for air quality (CONAMA Decree No. 03 of 06/28/90).

**Table 2:**
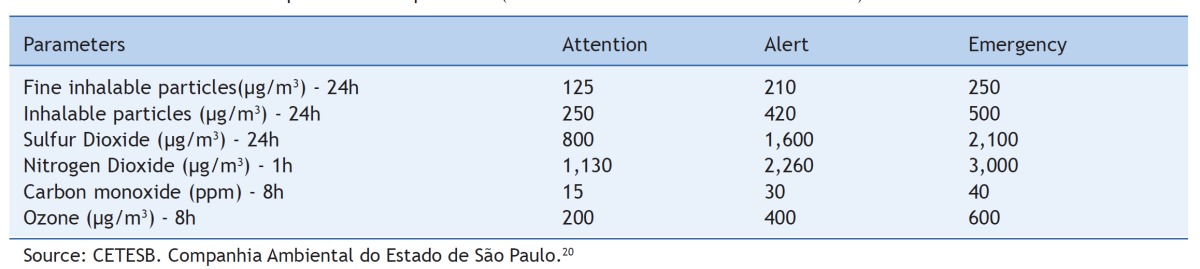
Criteria for acute episodes of air pollution (State Decree No. 59113 of 04/23/2013).

Daily measurements of pollutants are available at www.cetesb.sp.gov.br and, based on these values, the mean concentrations for the third trimester were calculated, for the last two months, and for the last month of pregnancy. Data analysis was primarily descriptive, and to assess the association between maternal exposure to air pollution and low birth weight, linear (univariate and multivariate) and logistic regression (univariate and multivariate) were used. In the linear regression, the variable outcome, birth weight in grams, was analyzed continuously, whereas in the logistic regression, birth weight was dichotomized (weight <2,500g and weight ≥2,500g). 

Regarding maternal exposure to pollutants, the linear regression considered the mean concentration, in the third trimester and monthly (each month of the third trimester) of each pollutant in the respective trimester of gestation, whereas in the logistic regression, these means were recoded into quartiles. The choice of the three month-period to estimate maternal exposure to air pollution was based on the fact that many studies^4^^,^ 6 ^-^ 8 ^,^ 21 that assessed pregnancy outcomes used the trimester as a unit measurement, especially the third. From a physiological viewpoint, during pregnancy, the velocity of fetal weight gain has a maximum growth period between the 28^th^ and 37^th^ weeks of gestation.^22^

The linear and logistic univariate analyses first assessed the association of birth weight and low birth weight, respectively, with maternal exposure to several pollutants, aiming to estimate the gross effect, i.e., without adjustments, of this exposure on the child's weight. Furthermore, the univariate logistic model was used to investigate the association between outcome and each variable found in the live birth certificates (LBCs; variables: maternal marital status, type of delivery, and gender of the newborn), in order to identify possible confounding factors. In this case, the statistical analysis was based on the calculation of the OR to estimate the risk of newborns with low birth weight associated with each variable. All analyses included 95% confidence intervals and significance level was set at 5% (α=5%). 

Based on the results of the univariate logistic model, variables in the multivariate models were selected, in order to control for confounders. First, the multivariate model (linear and logistic) was considered without the pollutant, i.e., the exposure of interest. The independent variables were entered one by one in both the linear and logistic regression, using the smallest *p*-value as the input criteria, provided that this was less than 0.25. If the variables showed *p*<0.001, the variable entry criteria started from the highest value of the OR observed in the univariate logistic analysis.

Furthermore, to verify the importance of each variable for the model and its permanence in it, the likelihood ratio was used, and only variables with *p*<0.05 remained in the final analysis. Only after obtaining the complete model were the pollutants included individually, and their association was tested with birth weight and low birth weight. Statistical analysis was performed using STATA v.7 (StataCorp LP - Texas, United States). 

The study project was approved by the Research Ethics Committee of Universidade de Taubate CEP/UNITAU No. 363/11.

### Results 

Of a total of 45,412 live births in the period of 2005 to 2009, 21,591 births were studied, considering the inclusion criteria described in the method section. Of the included newborns, it was observed that 13,149 (61.2%) were born to mothers who had partners, 14,575 (67.5%) were delivered by cesarean section, and 11,012 (51%) were males (Table 3). The frequency of LBW in the total of live births between 1,000g and 4,500g was 647 (3.0%). As for the mean and the median birth weight, the values were 3,237g and 3,225g, respectively. 

**Table 3:**
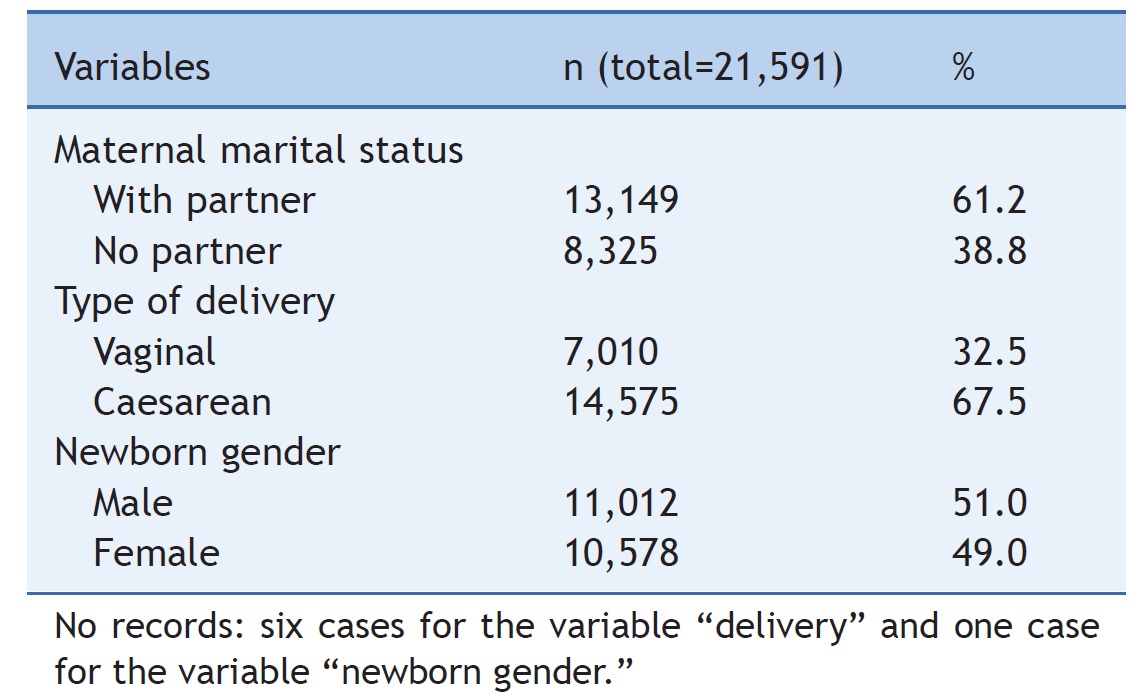
Distribution of live births to mothers residing in São José dos Campos-SP, from 2005 to 2009, according to maternal marital status, type of birth, and newborn gender

Table 4 shows the prevalence and the chance of low birth weight (LBW) among live births in São José dos Campos-SP according to maternal marital status, type of delivery, and gender of the newborn. The chance of a child being born weighing <2,500g was higher for mothers without a partner (OR 1.26, 95% CI: 1.08-1.48) and for female newborns (OR 1.65, 95% CI: 1.41-1.94). As for cesarean delivery, no statistical significance was observed (*p*=0.979). 

**Table 4:**
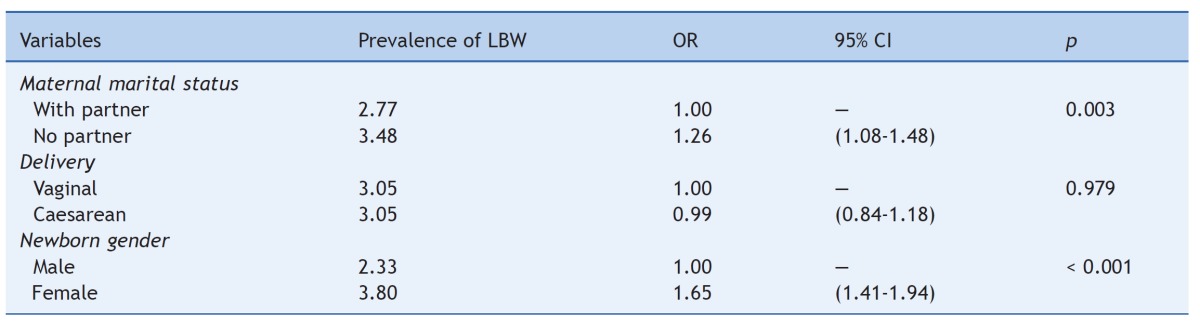
Prevalence and odds ratios (OR) with corresponding 95% confidence intervals (95% CI) for low birth weight (LBW) of live births in São José dos Campos-SP to mothers residing in the city between 2005 and 2009 according to maternal marital status, type of birth, and newborn gender.

Table 5 shows the descriptive analysis of the daily levels of air pollutants. The means of the pollutants are within acceptable air quality standards established in the CONAMA Resolution. However, PM_10_ and O_3_ had daily maximum values that were higher ​​than the acceptable values, 171 and 209mg/m^3^, respectively, which fits the "inadequate" and "bad" air quality classifications, respectively. These results are of concern and deserve attention. 

**Table 5:**
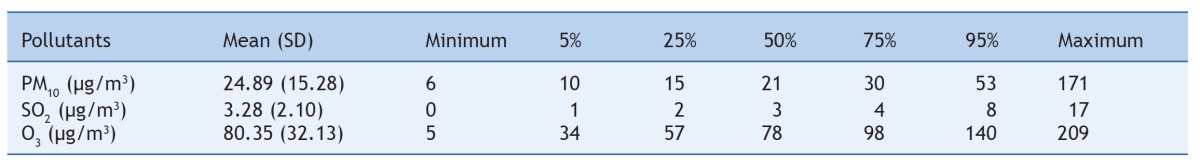
Descriptive analysis of the daily levels of air pollution in São José dos Campos between 2005 and 2009.

Maternal exposure to each pollutant was analyzed in quartiles and multiple logistic regression was performed using the last three months of pregnancy. In this analysis, adjustment was made for the variables maternal marital status and gender of the newborn. None of the pollutants showed statistically significant result, with the exception of SO_2_ in the last month, indicating that maternal exposure during this period is associated with a 1.25-fold greater chance of having a newborn with low birth weight (OR 1.25, 95% CI: 1.00 -1.56) for the second quartile of pollutant concentrations. 

Table 6 shows the odds ratios and respective confidence intervals for the presence of low birth weight according to quartiles of concentration of air pollutants for the last trimester, last two months, and last month of pregnancy in the studied population. During the last month of pregnancy, exposure to the three air pollutants indicated greater frequency of low birth weight, but without statistical significance. With the increase in the concentration of pollutants, represented in quartiles, no increase in the chance of outcome occurrence was observed. 

**Table 6:**
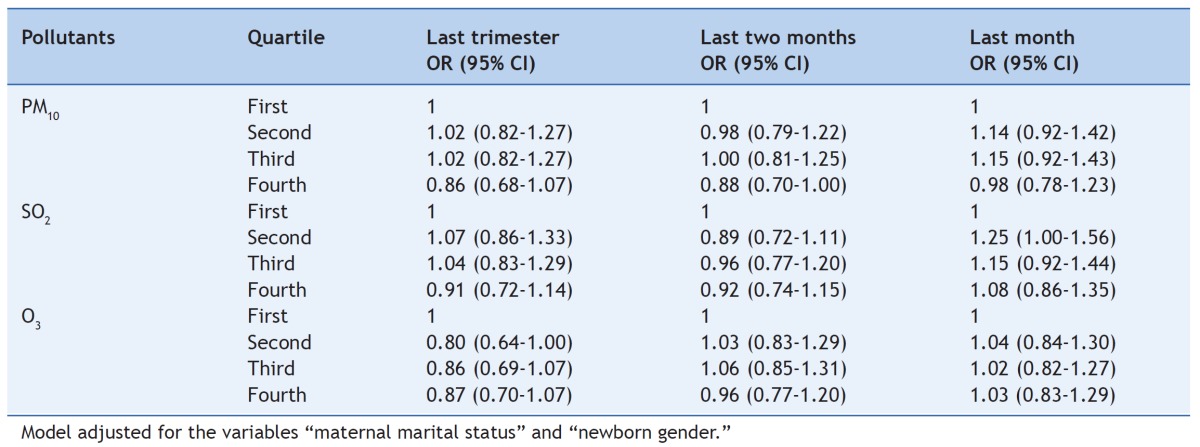
Odds ratio (OR) and confidence intervals (95% CI) for low birth weight (LBW) according to quartiles of concentration of air pollutants for the last trimester, last two months, and last month of pregnancy, in São José dos Campos between 2005 and 2009 (logistic regression).

Table 7 shows the coefficients of linear regression for birth weight according to the means of the last month, last two months, and last trimester of the corresponding concentration of air pollutants for the last trimester, last two months, and last month of pregnancy, adjusted for maternal marital status and gender of the newborn. It can be observed that in the last month, maternal exposure to PM_10_ and SO_2_ led to a decrease in mean birth weight: there was a decrease in weight of 3.8g and 38.6g for increases in mean maternal exposure of 10μg /m^3^ of PM_10_ and SO_2_, respectively. 

**Table 7:**
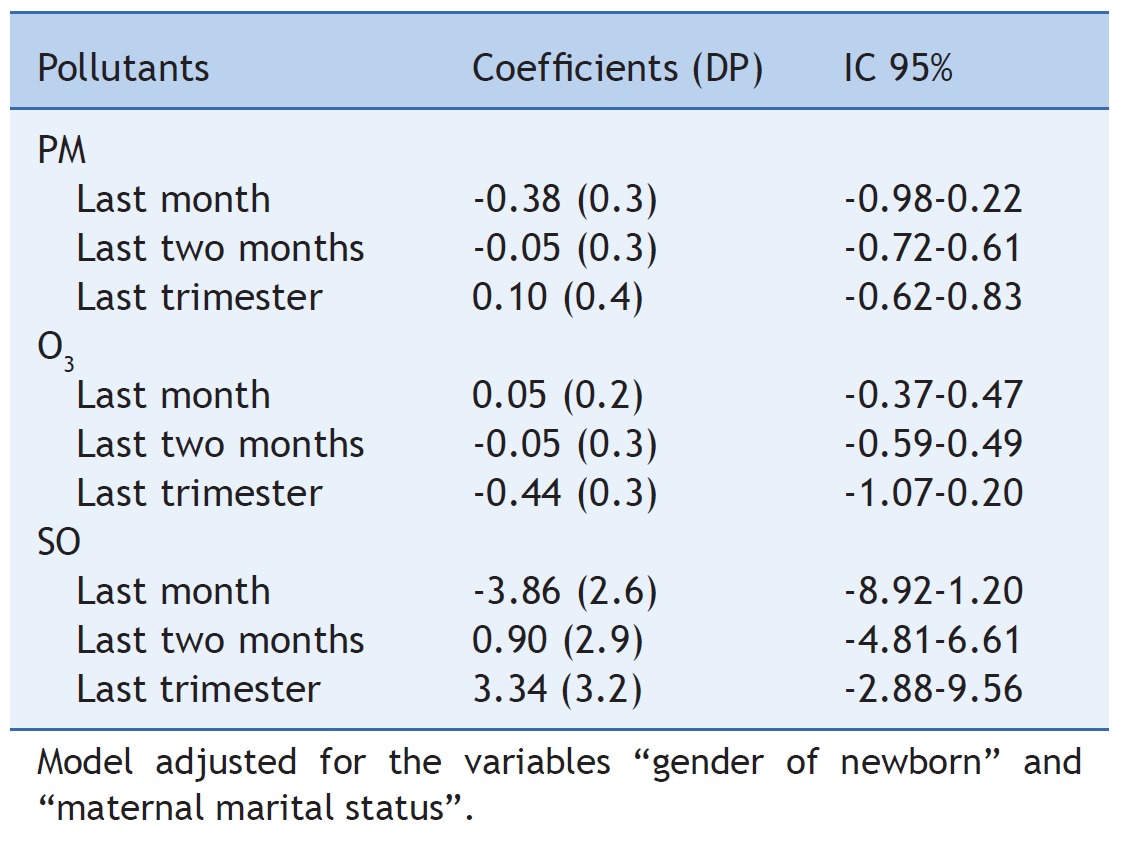
Regression coefficients with standard deviations (SD) and 95% confidence intervals (95% CI) according to the means of the last month, last two months, and last trimester of concentration of atmospheric pollutants corresponding to the last trimester, last two months, and last month of pregnancy, in São José dos Campos between 2005 and 2009 (multiple linear regression).

Exposure to O_3_ also appears to indicate a decrease in mean birth weight in the last trimester of pregnancy and the last two months. However, no result showed statistical significance. 

## Discussion

Low birth weight was associated with maternal marital status and gender of the newborn. Despite the predominance of male births, the prevalence of underweight was higher in female infants. Gouveia *et al* 7 also identified an increased risk for birth weight <2,500g in female newborns (OR 1.22, 95% CI: 1.20-1.24). This tendency for birth weight distribution of males to higher values when ​​compared to females was also observed in other studies, such as that by Areno^23^ and Tanaka.^24^

The study found a significant association between birth weight and maternal marital status, i.e., mothers living without a partner showed higher risk of having children with low birth weight. These results are consistent with those found by Miller *et al*,^25^ in the city of São Paulo, in which they estimated that the relative risk of LBW increased by 2.19-fold for mothers living without a partner in relation to mothers with a partner. 

Regarding the type of delivery, cesarean birth appears with a lower chance of insufficient weight; however, there was no statistically significant result in the present study. Antonio *et al* 26 observed similar results in their study, and hypothesized that as lower weight gain may be associated with a lower socioeconomic level, women belonging to this socioeconomic stratum are commonly users of the Unified Health System (Sistema Único de Saúde - SUS) in Brazil, which imposes limitations on the performance of cesarean deliveries, justifying a lower chance of underweight (2,500g to 2,999g) in cesarean births. According to Carniel *et al*,^27^ there is a higher cesarean section rate in groups of low obstetric risk and women of higher socioeconomic status, suggesting that the criteria for the indication of this procedure are not purely technical.

There was no statistical significance in the analyses conducted with atmospheric pollutants; however, the multiple logistic analysis showed that maternal exposure to SO_2_ in the last month of pregnancy poses an increased risk for low birth weight. The study by Reis^28^ showed a frequency of 9.1% for LBW, and risk of this outcome after exposure to SO_2_, PM_10_, and O_3_ in the second and third trimesters of pregnancy, even when the levels of pollutants found were below the standards established for air quality, i.e., a grade that represents good air quality (Table 3). Romao *et al* 29 also identified, in a population with 6% ​​prevalence of low birth weight, an association between this outcome and maternal exposure to PM_10_ (fourth quartile) in the quartile trimester of pregnancy. 

It can be observed that SO_2_ and PM_10_ were not associated with birth weight, which may result from the low frequency of newborns with low birth weight (3%), adding to that the fact that the mean concentrations of pollutants did not reach very high values​​, although the maximum values were inadequate according to air quality standards.

Regarding the study limitations, it is worth mentioning that, unlike the exposure data related to newborns, maternal factors, prenatal factors, and childbirth, which are obtained per individual, i.e., directly, the data regarding the exposure to air pollutants are obtained by indirect measurement by means of the concentration of air pollutants in the environment, which may hamper the consolidation of more consistent data. Another limitation refers to obtaining secondary data from DATASUL, an invaluable data source, but without the direct control of the researcher.

Although the results were not statistically significant and despite the difficulty to isolate the effect of each pollutant due to the high correlation among them, it was observed that SO_2_ can lead to low birth weight in the last month of maternal exposure. 
