# Supplementary material for: The effect of air pollutants on birth weight in medium-sized towns in the state of São Paulo
Source: Rev Paul Pediatr. 2014 Dec;32(4):306–12. doi: 10.1016/j.rpped.2014.06.003 (PMC4311783; doi:10.1016/j.rpped.2014.06.003)
Supplement: Translation

# Translation: O papel dos poluentes atmosféricos sobre o peso ao nascer em cidade de médio porte Paulista☆

**Authors:** Veridiana de Paula Santos, Andréa Paula Peneluppi de Medeiros, Thaiza Agostini Córdoba de Lima, Luiz Fernando Costa Nascimento

**Affiliations:** Universidade de Taubaté (UNITAU), Taubaté, SP, Brasil

**Keywords:** Baixo peso ao nascer, Poluição do ar, Regressão logística, Regressão linear

## Abstract

**OBJETIVO:**

Verificar o efeito da poluição do ar sobre o peso ao nascer numa cidade de médio porte paulista.

**MÉTODOS:**

Estudo transversal, com dados relativos a todos os nascidos vivos de mães residentes no Município de São José dos Campos nos anos de 2005 a 2009. Foram obtidos dados do Departamento de Informações e Informática do Sistema Único de Saúde. Os dados dos poluentes do ar (PM_10_, SO_2_ e O_3_), as médias diárias de suas concentrações, foram fornecidos pela Companhia de Tecnologia de Saneamento Ambiental. Aplicou-se a regressão linear e a logística para a análise dos dados, realizadas nos programas Excel e STATA v.7.

**RESULTADOS:**

A exposição materna aos poluentes do ar não se associou ao nascimento de crianças com baixo peso, com exceção do SO_2,_ no último mês de gestação (OR=1,25; IC95% 1,00-1,56). Além disso, a exposição materna ao PM_10_ e SO_2_ no último mês levou à diminuição do peso ao nascer (0,28g e 3,15g, respectivamente) para cada 1mcg/m^3^ de aumento da concentração desses poluentes, porém sem significância estatística.

**CONCLUSÕES:**

Este estudo não permitiu identificar associação estatística entre os níveis de concentração dos poluentes atmosféricos e o peso ao nascer, com exceção da exposição SO_2,_ no último mês de gestação.

## Introdução

A poluição do ar representa hoje um dos maiores problemas de Saúde Pública, afetando a saúde dos seres humanos, animais e das plantas. O rápido avanço tecnológico do mundo moderno trouxe consigo um aumento na quantidade e na variedade de poluentes eliminados na atmosfera, prejudicando a qualidade de vida em nosso planeta.^1^ Os principais poluentes atmosféricos nas cidades são o material particulado (PM_10_), o ozônio (O_3_), o dióxido de enxofre (SO_2_), o monóxido de carbono (CO) e os óxidos de nitrogênio (NO_2_).

A exposição aos poluentes do ar vem se mostrando associada a vários efeitos deletérios sobre a saúde da população, mesmo em níveis considerados seguros pela legislação ambiental.^1^^,^ 2 Quando é medida a concentração dos poluentes atmosféricos de uma determinada localidade, identifica-se que as maiores concentrações produzem efeitos adversos à saúde, como o aumento no número de internações hospitalares, o aumento da mortalidade e a diminuição da expectativa de vida.^3^ Os efeitos da poluição do ar sobre desfechos relacionados com a gravidez também têm sido considerados em alguns estudos.^4^^-^ 6 Entre esses desfechos está o baixo peso ao nascer (BPN),^7^^,^ 8 definido como o nascido vivo com peso menor que 2500g.^9 ^Os mecanismos biológicos envolvidos no comprometimento do crescimento fetal associado à poluição ambiental parecem relacionados às alterações placentárias, com alterações anatomopatológicas e morfométricas,^10^ infarto placentário^11^ e vilosite crônica.^12^ Pesquisa realizada por Perera *et al* em gestantes dominicanas e afro-americanas entre 18 e 35 anos de idade residentes há pelo menos um ano em Nova York, não fumantes, sem diabetes, hipertensão arterial e soronegativas para o vírus da imunodeficiência humana indicou que, na população estudada, o feto e o recém-nascido são mais suscetíveis que os adultos às substâncias tóxicas ambientais.^13^

O peso ao nascer é um importante determinante da morbimortalidade neonatal e da mortalidade pós-neonatal,^14^ sendo assim de grande importância para a saúde pública. Dessa forma, a Organização Mundial da Saúde (OMS) considera o BPN como o fator isolado mais importante na sobrevivência infantil. Crianças com baixo peso ao nascer apresentam risco de mortalidade significativamente superior ao de crianças nascidas com peso ≥2.500 g.^15^ O BPN está presente em 15,5% de todos os nascimentos ocorridos no mundo. No entanto, o problema não ocorre de maneira uniforme entre as diversas localidades, mas se relaciona à situação socioeconômica que as mesmas apresentam. A maior porcentagem de crianças com esse agravo concentra-se em duas regiões do mundo, Ásia e África, com respectivamente 27% e 22% de todos os nascidos vivos com baixo peso.^16^ Em países desenvolvidos, de modo geral a proporção de BPN situa-se entre 4% e 6%.^17^ Em 2008, o Brasil apresentou uma proporção de 8,3% e o município de São José dos Campos de 9,1%.^18^

O BPN tem sido alvo de vários estudos epidemiológicos^4^^,^ 7 ^,^ 8 ^,^ 15 com o objetivo de identificar os seus fatores de risco, na tentativa de elaborar intervenções que possam reduzir estes fatores e prevenir sua ocorrência. A importância do BPN para a Saúde Pública é determinada não apenas pelos riscos subsequentes de mortalidade e morbidade, mas também pela frequência com que o mesmo ocorre. Nesse contexto, o presente estudo tem como objetivo avaliar o efeito da poluição do ar sobre o peso ao nascer de recém-nascidos de mães residentes no Município de São José dos Campos, nos anos de 2005 a 2009.

## Método

Trata-se de estudo transversal, com dados relativos a todos os nascidos de mães residentes no Município de São José dos Campos nos anos de 2005 a 2009 que tiveram sua ficha de declaração de nascido vivo preenchida. 

São José dos Campos situa-se a 80km de São Paulo, entre as serras do Mar e Mantiqueira, e conta com população de aproximados 700 mil habitantes. Sua altitude é de 600m acima do nível do mar e geograficamente está na latitude 23°11' sul e 45° 53' longitude oeste. Conta com cerca de 1.100 estabelecimentos industriais destacando-se montadoras de automóveis, indústrias farmacêutica e aeronáutica e refinaria de petróleo. 

As informações sobre os nascidos vivos foram obtidas por meio de consulta à base de dados do Sistema de Informações sobre Nascidos Vivos (SINASC) por meio da Declaração de Nascido Vivo (DN)¸disponibilizados pelo Departamento de Informática do Sistema Único de Saúde (DATASUS), no endereço eletrônico (http://www.datasus.gov.br). Incluíram-se os recém-nascidos à termo, com peso entre 1000g e 4500g, de gestação única, com idade materna entre 20 e 34 anos, sete ou mais consultas de pré-natal e escolaridade materna de 8 ou mais anos de estudo concluído. Esses critérios foram adotados para se eliminar situações de maior vulnerabilidade como o recém-nascido pré-termo e o peso abaixo de 1.000g, para os quais outros fatores de risco poderiam ser mais relevantes do que a poluição atmosférica.

Os poluentes estudados foram o PM_10_, SO_2_ e O_3_, que são quantificados diariamente pela estação medidora da Companhia de Tecnologia de Saneamento Ambiental (CETESB) de São José dos Campos. No Brasil os padrões de qualidade do ar foram estabelecidos pela Resolução CONAMA nº 3/1990^19^ (tabela 1), no entanto, no estado de São Paulo houve a publicação do Decreto Estadual nº 59113 de 23/04/2013,^20^ estabelecendo novos padrões de qualidade do ar (tabela 2). As medidas diárias dos poluentes foram disponibilizadas no endereço eletrônico (www.cetesb.sp.gov.br) e, a partir desses valores, foram calculadas as médias das concentrações referentes ao 3º trimestre, dos últimos dois meses e do último mês de gestação. A análise dos dados foi primeiramente descritiva e, para examinar a associação entre exposição materna à poluição do ar e o BPN, foram utilizadas a regressão linear (univariada e multivariada) e a logística (univariada e multivariada). Na regressão linear, a variável desfecho, peso ao nascer em gramas, foi analisada de forma contínua, enquanto que, na regressão logística, o peso ao nascer foi dicotomizado (peso<2500g e peso ≥2500g).

**Tabela 1:** Padrões nacionais de qualidade do ar (Resolução CONAMA no. 3, de 28/06/1990)

**Tabela 2:** Critérios para episódios agudos de poluição do ar (Decreto Estadual no. 59.113, de 23/04/2013)

Com relação à exposição materna aos poluentes, na regressão linear foram consideradas a média do 3º trimestre e mensal (dos meses do 3º trimestre) da concentração de cada poluente no respectivo trimestre de gestação, enquanto, na regressão logística, tais médias foram recodificadas em quartis. A escolha do período de três meses para a estimativa da exposição da mãe à poluição do ar se baseou no fato de que muitos estudos,^4^^,^ 6 ^-^ 8 ^,^ 21 que avaliam os desfechos da gravidez, utilizam o trimestre de gestação como unidade de mensuração, principalmente o terceiro trimestre. Do ponto de vista fisiológico, durante a gravidez, a velocidade do ganho de peso fetal apresenta um período de crescimento máximo que ocorre entre 28^a^ e a 37^a^ semana de gestação.^22^

As análises univariadas linear e logística examinaram, primeiramente, a relação do peso e do baixo peso ao nascer, respectivamente, com a exposição materna aos diversos poluentes com o objetivo de estimar o efeito bruto, ou seja, sem ajustes, dessa exposição no peso da criança. Além disso, o modelo logístico univariado foi utilizado para verificar a relação do desfecho com cada variável presente na Declaração de Nascido Vivo (DNV; variáveis: estado civil materno, tipo de parto e sexo do recém-nascido), de maneira a identificar possíveis fatores de confusão. Neste caso, a análise estatística baseou-se no cálculo do OR para se estimar o risco de recém-nascido com baixo peso ao nascer associado a cada variável. Em todas as análises foram construídos intervalos de 95% de confiança e adotado nível de significância de 5% (α=5%).

 A partir dos resultados deste modelo logístico univariado, foram selecionadas as variáveis para os modelos multivariados, com a finalidade de se fazer o controle de confusão. Primeiramente o modelo multivariado (linear e logístico) foi considerado sem o poluente, ou seja, a exposição de interesse. As variáveis independentes entraram uma a uma, tanto na regressão linear quanto na logística, utilizando-se como critério da ordem de entrada o menor valor do "p", contanto que este fosse menor que 0,25. Caso as variáveis apresentassem *p<*0,001, o critério da entrada das variáveis partiu do maior valor do OR observado na análise logística univariada. E ainda, para verificação da importância de cada variável para o modelo e sua permanência neste, utilizou-se a razão de verossimilhança (likelihood-ratio test), permanecendo no final da análise apenas as variáveis com *p<*0,05. Apenas após obter o modelo completo, os poluentes foram incluídos individualmente e testou-se sua associação com o peso ao nascimento e o baixo peso ao nascer. A análise estatística foi realizada no STATA v.7. 

O projeto deste estudo foi avaliado e aprovado pelo Comitê de Ética da Universidade de Taubaté CEP/UNITAU n^o^ 363/11.

## Resultados

Do total de 45.412 nascidos vivos do período entre 2005 e 2009, foram estudados 21.591 nascimentos, considerando-se os critérios de inclusão apresentados na metodologia. Destes incluídos, observou-se que 13.149 (61,2%) eram filhos de mães que possuíam parceiro, 14.575 (67,5%) nasceram de parto tipo cesáreo e 11.012 (51%) eram do sexo masculino (tabela 3). A frequência de BPN sobre o total de nascidos vivos com peso entre 1000g e 4500g foi de 647 (3,0%). Quanto à média e à mediana do peso ao nascer, os valores foram, respectivamente, 3.237 g e 3.225g.

**Tabela 3:** Distribuição dos nascidos vivos de mães residentes em São José dos Campos-SP no período entre 2005 e 2009, segundo estado civil materno, tipo de parto e sexo do recém-nascido

A tabela 4 mostra a prevalência e a chance de baixo peso ao nascer (BPN) dos nascidos vivos no município de São José dos Campos-SP segundo estado civil materno, tipo de parto e sexo do recém-nascido. A chance de a criança nascer com peso abaixo de 2500g foi maior nas mães sem parceiro (OR=1,26; IC95% 1,08-1,48) e nos recém-nascidos do sexo feminino (OR=1,65; IC95% 1,41-1,94). Já para o parto cesáreo, não se observou significância estatística (*p=*0,979). 

**Tabela 4:** Prevalência e odds ratio (OR) com respectivos intervalos de 95% de confiança (IC95%) de baixo peso ao nascer (BPN) dos nascidos vivos no município de São José dos Campos-SP de mães residentes nesta cidade no período entre 2005 e 2009, segundo estado civil materno, tipo de parto e sexo do recém-nascido

A tabela 5 apresenta a análise descritiva dos níveis diários dos poluentes atmosféricos. As médias dos poluentes estão dentro dos padrões aceitáveis de qualidade do ar, estabelecidos na resolução CONAMA (Conselho Nacional de Meio Ambiente). No entanto, os poluentes PM_10_ e O_3_ tiveram seus valores máximos diários maiores do que os valores aceitáveis, respectivamente, 171 e 209 µg/m^3^, enquadrando-se na classificação "inadequada" e "má", respectivamente. Esses resultados são preocupantes e merecem atenção.

**Tabela 5:** Análise descritiva dos níveis diários de poluição do ar no município de São José dos Campos entre 2005 e 2009

A exposição materna a cada poluente foi analisada em quartis e realizada regressão logística múltipla segundo os últimos três meses de gestação. Nesta análise, houve o ajuste para as seguintes variáveis: estado civil materno e sexo do recém-nascido. Nenhum poluente apresentou resultado estatisticamente significante, com exceção do SO_2 _no último mês, indicando que a exposição materna nesse período se associa à 1,25 vezes mais chance de recém-nascido de baixo peso (OR 1,25; IC 95% 1,00-1,56) para o 2º quartil da concentração do poluente.

A tabela 6 mostra a razão de chances e seu intervalo de confiança para a presença de baixo peso ao nascer de acordo com os quartis de concentração dos poluentes atmosféricos para o último trimestre, último bimestre e último mês de gestação, na população analisada. Durante o último mês gestacional, a exposição aos três poluentes atmosféricos indicou maior chance de ocorrência de baixo peso ao nascer, mas sem significância estatística. Com o aumento da concentração dos poluentes, representada em quartis, não se observou aumento da chance de ocorrência do desfecho.

**Tabela 6:** Odds ratio (OR), intervalos de confiança a 95% (IC95%) para baixo peso ao nascer (BPN) de acordo com os quartis de concentração dos poluentes atmosféricos para o último trimestre, último bimestre e último mês de gestação, no município de São José dos Campos entre 2005 e 2009 (regressão logística múltipla)

Já a tabela 7 indica os coeficientes de regressão linear para o peso ao nascer segundo as médias do último mês, último bimestre e último trimestre da concentração dos poluentes atmosféricos correspondentes para o último trimestre, último bimestre e último mês de gestação, ajustados para estado civil materno e sexo do recém-nascido. Pode-se verificar que, no último mês, a exposição materna ao PM_10_ e ao SO_2_ levou a uma diminuição da média do peso ao nascer: houve decréscimo do peso de 3,8g e 38,6gpara um aumento na exposição média materna de, respectivamente, 10μg/m^3 ^do PM_10 _e do SO_2_. A exposição ao O_3_ também parece indicar essa diminuição da média do peso ao nascer no último bimestre e trimestre de gestação. No entanto, nenhum resultado amostrou significância estatística.

**Tabela 7:** Coeficientes de regressão com seus respectivos desvios-padrões (DP) e intervalos de confiança de 95%, segundo as médias do último mês, último bimestre e último trimestre da concentração dos poluentes atmosféricos correspondentes para o último trimestre, último bimestre e último mês de gestação, no município de São José dos Campos entre 2005 e 2009 (regressão linear múltipla)

## Discussão

O baixo peso ao nascer esteve associado ao estado civil materno e ao sexo do recém-nascido. Apesar do predomínio de nascimentos do sexo masculino, a prevalência de baixo peso foi maior nas crianças do sexo feminino. Gouveia *et al* 7 também identificaram um maior risco para peso ao nascer abaixo de 2500g em recém-nascidos femininos (OR=1,22; IC 95%: 1,20-1,24). Esta tendência da distribuição do peso ao nascer da população masculina para valores maiores em comparação à feminina também foi observada em outros estudos como o de Areno^23^ e Tanaka.^24^

No estudo encontrou-se associação significativa com o peso ao nascer e o estado civil materno, ou seja, mães vivendo sem o parceiro mostram risco maior de terem filhos com baixo peso ao nascer. Estes resultados são consistentes com os encontrados por Monteiro *et al*,^25^ para a cidade de São Paulo, em que calcularam que o risco relativo de BPN aumentava 2,19 para mães vivendo sem parceiro em relação a mães com parceiro.

Com relação ao tipo de parto, o cesáreo aparece com menor chance de ocorrência de peso insuficiente; no entanto, não se encontrou resultado estatisticamente significante no presente estudo. Antonio *et al* 26 observaram, em seu estudo, resultados semelhantes e supôs que, como o menor ganho de peso pode estar relacionado com um pior nível socioeconômico, as mulheres pertencentes a este estrato são comumente usuárias do Sistema Único de Saúde, o que impõe limitações para a ocorrência de partos cesáreos, justificando uma menor chance de peso insuficiente (2500g a 2999g) entre os partos cesáreos . Segundo Carniel *et al*,^27^ há uma maior ocorrência de cesarianas entre grupos de baixo risco obstétrico e de mulheres com melhores condições sociais, sugerindo que os critérios para indicação desse procedimento não são exclusivamente técnicos. 

Não se encontrou significância estatística nas análises realizadas com os poluentes atmosféricos, porém, foi observado na análise logística múltipla que a exposição materna ao SO_2_ durante o último mês gestacional apresenta um maior risco para o nascimento de uma criança com baixo peso. No estudo de Reis,^28^ verificou-se uma frequência de 9,1% de BPN e risco desse desfecho diante da exposição ao SO_2_, PM_10_ e O_3_ no 2º e 3º trimestres de gestação, mesmo quando os níveis das concentrações dos poluentes se encontravam abaixo dos padrões estabelecidos para a qualidade do ar, ou seja, com "nota" que representa boa qualidade do ar (tabela 1). Romão *et al* 29 também identificaram, em população com 6% de prevalência de BPN, associação desse desfecho com a exposição materna ao PM_10_ (4º quartil) no 3º trimestre de gestação.

Pôde-se observar que o SO_2_ e PM_10_ não mostraram associação com o peso do RN, o que pode decorrer da baixa frequência de recém-nascidos com baixo peso ao nascer (3%), somando-se a isso as médias das concentrações dos poluentes não atingiram valores muito altos, apesar dos valores máximos serem inadequados segundo os padrões da qualidade do ar.

Com relação às limitações deste estudo, vale comentar que, diferentemente dos dados de exposição relativos ao recém-nascido, à mãe, ao parto e pré-natal, que se obtém indivíduo a indivíduo, ou seja, de forma direta, os dados referentes à exposição aos poluentes do ar são obtidos por medida indireta, por meio da concentração dos poluentes do ar no ambiente, o que pode dificultar a consolidação de dados mais consistentes como os individuais. Outra limitação a ser apontada refere-se à obtenção de dados secundários do DATASUS, fonte valiosa de dados, porém sem o controle direto do pesquisador como se tem com a coleta de dados de fontes primárias.

Apesar dos resultados não serem estatisticamente significantes e da dificuldade em isolar o efeito de cada poluente, devido à alta correlação entre eles, observou-se que o SO_2_ pode levar ao baixo peso ao nascer, no último mês de exposição materna.
